# From risk factors to molecular targets: clinical associations and molecular docking insights into phthalate-associated diabetic retinopathy

**DOI:** 10.3389/fmed.2026.1792532

**Published:** 2026-05-13

**Authors:** Zhiwei Xu, Shi Bai, Caidi He, Xiaobei Lv, Qin Li, Qile Mao, Haijian Wu, Peter Wang

**Affiliations:** 1 Department of Ophthalmology, Taizhou Municipal Hospital, Taizhou University Affiliated Municipal Hospital, Taizhou, Zhejiang, China; 2 School of Medicine, Taizhou University, Taizhou, Zhejiang, China; 3 Department of Medicine, Beijing Zhongwei Medical Research Center, Beijing, China; 4 Department of Biology, Oasis Road Research Center, Watertown, MA, United States

**Keywords:** bioinformatics, DEHP, diabetes, environmental exposure, NHANES, Phthalate, retinopathy

## Abstract

**Objective:**

This study aimed to identify novel risk factors for diabetic retinopathy (DR) and to explore whether phthalate exposure contribute to DR pathogenesis.

**Methods:**

We analyzed data from the 2017–2018 National Health and Nutrition Examination Survey (NHANES) and an independent clinical cohort using multiple feature-selection methods. To investigate the potential mechanisms of di-2-ethylhexyl phthalate (DEHP), we performed network toxicology and molecular docking analyes.

**Results:**

In NHANES, longer weekday outdoor time was associated with higher odds of DR (OR = 3.18, 95% CI: 1.36–7.95), whereas higher serum epi-25-hydroxyvitamin D3 levels were associated with lower odds of DR (OR = 0.80, 95% CI: 0.64–0.97). In the clinical cohort, less electronic device use was correlated with lower odds of DR (OR = 0.67, 95% CI: 0.43–0.98), while cataract was strongly associated with DR (OR = 11.0, 95% CI: 1.77–99.8). Urinary phthalate assessment showed inverse associations of mono-isobutyl phthalate (MiBP) and a mixed phthalate index with DR. Network toxicology identified 123 overlapping target genes, with enrichment in AGE-RAGE signaling and apoptosis-related pathways. Molecular docking predicted stable binding of MiBP to SRC, BCL2, and MAPK1.

**Conclusion:**

This integrative analysis identifies novel clinical and environmental correlates of DR and suggests a possible link between phthalate exposure and DR-related pathways. These findings highlight the potential relevance of environmental exposures in DR risk and offer a basis for further mechanistic and preventive research.

## Introduction

Diabetic retinopathy (DR) is a sight-threatening microvascular complication of diabetes, resulting from chronic damage to the retinal vasculature (1, 2). Globally, approximately 22.27% of diabetic patients are affected by DR, indicating that roughly one in four patients is likely to develop this condition (3). The health burden of DR is projected to rise significantly, with the number of affected individuals projected to increase from 103 million in 2020 to 160 million by 2045 (4, 5). Established determinants of DR include diabetes duration and long-term glycemic control (6). Without timely intervention, DR can progressively impair vision, leading to symptoms that range from mild blurriness to irreversible blindness (7). Early-stage DR is frequently asymptomatic, and clinically apparent signs may not emerge until substantial visual impairment has occurred. While routine screening can improve early detection and reduce vision loss (8, 9), there remains an urgent need to identify additional, modifiable risk factors. Previous studies suggest that lifestyle behaviors, including regular physical activity and a balanced diet, may reduce DR risk (10, 11). Additionally, hypertension, elevated HbA1c levels, higher body mass index (BMI), and insufficient sleep have been correlated with DR development (12–16).

Emerging evidence suggests that vitamin D deficiency may increase the risk of DR among patients with type 2 diabetes (17, 18). In contrast, the relationship between electronic device use and DR remains incompletely characterized. A Mendelian randomization study reported no evidence that greater leisure screen time increases DR risk (19), while a prospective cohort study observed elevated hazard ratios (HR) for DR among participants using mobile phones for more than 1.5 h per day compared with those with shorter use (20). Beyond lifestyle and clinical characteristics, exposure to environmental chemicals represents an additional, increasingly important area for DR risk assessment. In a recent analysis of endocrine-disrupting chemicals (EDCs) and all-cause mortality in diabetic patients, bisphenol A (BPA) and mono-(3-carboxypropyl) phthalate (MCPP) were positively associated with mortality among participants with DR (21). Phthalate exposure occurs mainly through ingestion, dermal contact, and inhalation, and urinary metabolites—detected in the vast majority of urine samples—serve as reliable biomarkers for assessing exposure (22–25). Although phthalates have been linked to adverse metabolic outcomes and diabetic traits, their specific contribution to DR onset and progression remains inadequately studied.

To systematically investigate potential mechanistic links, we applied a network toxicology framework, which integrates multi-component regulatory networks to prioritize candidate toxic targets and pathways (12), together with molecular docking simulations to predict ligand-receptor interactions (26, 27). Together, these complementary approaches can help elucidate the molecular basis of chemical-associated toxicity. The present study aimed to identify novel risk factors associated with DR using the NHANES dataset and an independent, self-collected validation cohort. In parallel with the epidemiological analyses, we further explored the potential molecular mechanisms underlying phthalate-associated DR toxicity, thereby providing new insights into how environmental pollutants may contribute to this sight-threatening diabetic complication.

## Materials and methods

### Study population

**FIGURE 1:**
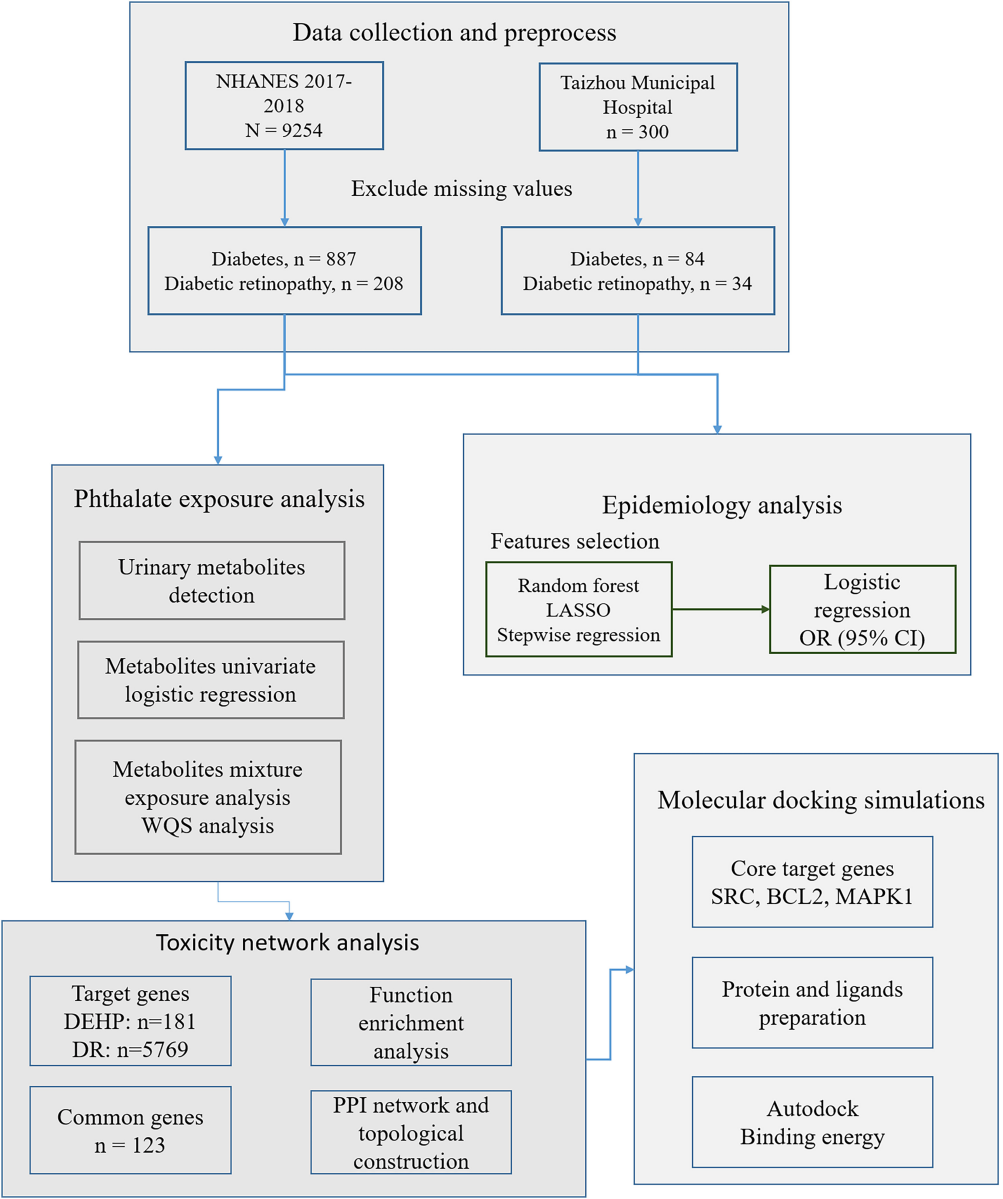
The workflow of this study.

This study employed data from the National Health and Nutrition Examination Survey (NHANES), an ongoing cross-sectional program conducted by the Centers for Disease Control and Prevention (CDC) and the National Center for Health Statistics (NCHS). From the 2017–2018 survey cycle, we included 887 adults with diabetes. Additionally, an independent validation cohort was established, comprising 300 patients recruited from the ophthalmology inpatient department of Taizhou Municipal Hospital between March and September 2025. The study evaluated associations between lifestyle and environmental variables—including time spent outdoors and electronic device use—and the presence of DR among individuals with diabetes (Figure 1).

### Assessment of covariates in clinical datasets

From the 9,254 participants in the 2017–2018 NHANES cohort, 887 diabetic patients were included based on self-reported physician-diagnosed diabetes and a valid response regarding retinopathy status. A total of 27 covariates were analyzed, encompassing gender (female, male), age, race/ethnicity (Mexican American, Other Hispanic, Non-Hispanic White, Non-Hispanic Black, Non-Hispanic Asian, Other Race–including Multi-Racial), education level (< 9th grade, 9–11th grade, high school graduate, some college, college graduate or above), history of hypertension (yes/no), high cholesterol (yes/no), skin reaction to sun exposure (severe sunburn with blisters, severe sunburn for several days with peeling, mild burn with some tanning, tanning without sunburn, no reaction after 30 min), frequency of staying in the shade (always, most of the time, sometimes, rarely), sunscreen use frequency (always, most of the time, sometimes, rarely, never), history of sunburn (yes/no), weekday outdoors time (≤ 14 min, > 14 min), weekend outdoor time (≤ 14 min, > 14 min), sedentary activity time (minutes), trouble sleeping (yes, no, don’t know), weight, body mass index (BMI), total cholesterol, vitamin D measures (25OHD2 + 25OHD3, 25OHD2, 25OHD3, epi-25OHD3), diastolic blood pressure, systolic blood pressure, glycated hemoglobin, annual household income (high, low), and triglycerides. Individuals with over 20% missing data across covariates were excluded from analysis. Missing values in covariates were imputed using multiple imputation by chained equations (MICE) implemented in the mice package in R, with the following parameters: m = 5, maxit = 50, and method = “PMM.”

An independent validation cohort was derived from Taizhou Municipal Hospital. Following the exclusion of patients with incomplete medical records, a total of 300 participants were included. Among them, 84 individuals had diabetes, including 34 diagnosed with DR. For the hospital cohort, the following variables were extracted from medical records and questionnaires: demographic characteristics (age, gender), anthropometric measures (weight, height), lifestyle factors (time spent outdoors on weekdays and weekends, shade-seeking behavior, intensive work, smoking history, alcohol consumption frequency, electronic device usage, sedentary time, sleep duration and quality), clinical history (hypertension, hyperlipidemia, diabetes diagnosis, diabetic symptoms, glaucoma, macular degeneration, cataract), and biochemical parameters (systolic and diastolic blood pressure, triglyceride levels, cholesterol, glucose, HbA1c). Based on the distribution of outdoor time, a cutoff of 30 min was applied to categorize exposure. Notably, the occupational profile of this cohort differed substantially from that of the NHANES population, with a predominance of agricultural workers, which likely contribute to the markedly longer outdoor exposure durations.

### Feature selection and model construction

For both the NHANES and clinical validation datasets, we applied three distinct feature selection methods: random forest, least absolute shrinkage and selection operator (LASSO), and stepwise regression. Default parameters were used for the random forest analysis, as implemented in the randomForest package in R. LASSO regression was performed using the glmnet package, with 10-fold cross-validation to select the optimal tuning parameter. Bidirectional stepwise regression was conducted using the Akaike information criterion (AIC). Variable importance derived from the random forest algorithm was visualized using bar plots. Although the three methods yielded generally consistent variable selections, stepwise regression produced a more interpretable model and provided clearer insight when adjusting for covariates. Consequently, stepwise regression was selected as the primary modeling strategy for subsequent analyses. The odds ratios of the resulting variables were presented using forest plots.

### Measurement of urinary phthalate metabolites

In the 2017–2018 NHANES cohort, urinary DEHP was detected in 2,986 participants. After integrating data availability for DR status, 271 individuals were included in the final analytical sample. To comprehensively assess phthalate exposure, we selected ten urinary metabolites as biomarkers, including: mono-2-ethyl-5-carboxypentyl phthalate (MECPP), mono-n-butyl phthalate (MnBP), mono-(2-ethyl-5-hydroxyhexyl) phthalate (MEHHP), mono-(2-ethyl-5-oxohexyl) phthalate (MEOHP), monoisobutyl phthalate (MiBP), monocarboxynonyl phthalate (MCNP), monocarboxyoctyl phthalate (MCOP), mono(3-carboxypropyl) phthalate (MCPP), monoethyl phthalate (MEP), and monobenzyl phthalate (MBzP).

### Acquisition of potential target genes

DEHP target genes were retrieved from ChEMBL (all results included), PharmMapper (normalized fit score > 0.7), and SwissTargetPrediction (top 100 targets). These three gene lists were merged, and duplicates were removed to generate a final set of unique DEHP-associated genes. DR-associated genes were obtained from TTD (all entries), DisGeNET (all entries), and GeneCards (relevance score > 1). These three lists were likewise merged, and duplicate entries were removed. Gene symbols were standardized using UniProt. Overlapping genes between the DEHP target set and DR gene set were visualized using a Venn diagram and carried forward as common targets for further analysis. A protein–protein interaction (PPI) network of the common targets was constructed using the STRING database with a high-confidence interaction threshold (confidence score ≥ 0.9). The network was imported into Cytoscape (v3.9.1) for visualization and topological analysis. Topological properties of nodes were evaluated with the CytoNCA plugin, and the top 14 genes with the highest degree values were defined as core targets for subsequent molecular docking.

### Functional enrichment analysis

Gene Ontology (GO) and Kyoto Encyclopedia of Genes and Genomes (KEGG) pathway enrichment analyses were conducted for the common target genes using the “clusterProfiler” and “org.Hs.eg.db” packages in R. For GO enrichment, the top 15 significantly enriched terms from each category—molecular function (MF), cellular component (CC), and biological process (BP)—were extracted for functional interpretation. The top 10 enriched KEGG pathways were visualized using dot plot. In addition, Metascape was utilized to identify functional modules and to cluster genes based on shared biological functions.

### Molecular docking simulations

Molecular docking was performed using AutoDock Vina (v1.1.2) to predict binding conformations and affinities between selected ligands and target proteins. Three-dimensional protein structures were obtained from the Protein Data Bank (PDB) and preprocessed with AutoDockTools (v1.5.7), including removal of water molecules, addition of polar hydrogens, and assignment of Kollman charges, before proteins were saved in PDBQT format. Ligand structures were energy-minimized and similarly converted to PDBQT format with Gasteiger charges assigned and rotatable bonds defined. For each target, a grid box was set to encompass the predicted binding site of each target. Docking poses were ranked by predicted binding affinity (ΔG, kcal/mol), and the most favorable conformation for each ligand was selected for interaction analysis.

### Statistical analysis

All statistical analyses were performed using R software (v4.2.1). Continuous variables are expressed as mean ± standard deviation (SD), and categorical variables are presented as unweighted counts and percentages. Group comparisons were conducted using linear regression for continuous variables and chi-square tests for categorical variables. Associations between potential covariates and DR were assessed using logistic regression within the generalized linear model framework, with results reported as odds ratios (ORs) and 95% confidence intervals (CIs). To address the left-skewed distribution of urinary phthalate metabolites, concentrations were log10-transformed to approximate normal distribution. Transformed values were then categorized into quartiles for subsequent analysis. Logistic regression was employed to evaluate associations between metabolite quartiles and DR using three sequential adjustment levels: Model 1 (unadjusted), Model 2 (adjusted for gender, race, and age), and Model 3 (further adjusted for education level and alcohol use).

## Results

### Assessment of the association in NHANES dataset

**FIGURE 2:**
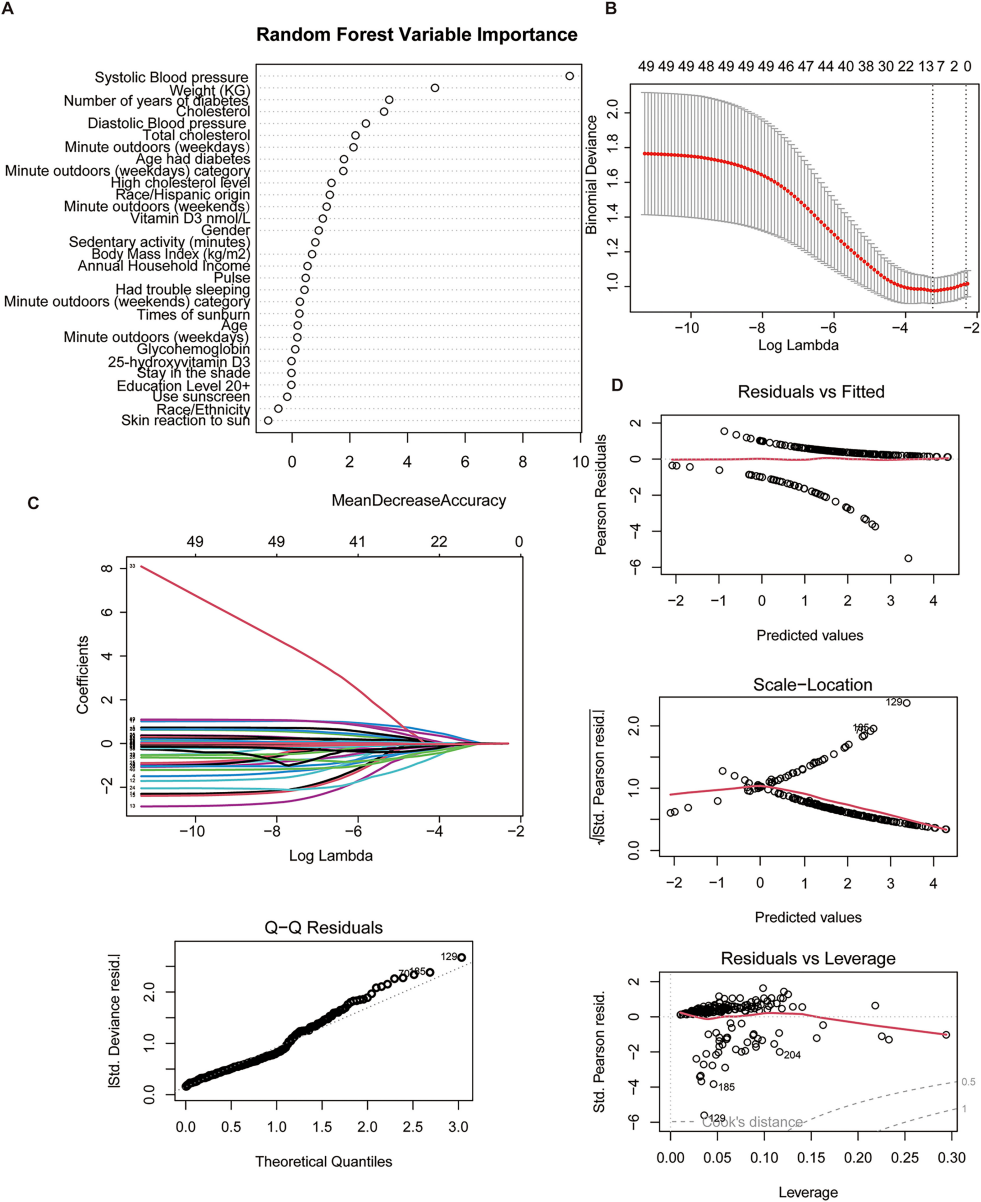
Feature selection with random forest and Lasso regression. **(A)** Variable importance from the random forest model. **(B)** LASSO coefficient profiles. **(C)** Cross-validation for selection of the LASSO tuning parameter. **(D)** Diagnostic plots for the LASSO regression model.

After data preprocessing, 210 diabetic patients were included, of whom 44 (21.0%) had retinopathy. The cohort was balanced in terms of gender distribution. Patients with DR were approximately 1 year older than those without DR. Baseline characteristics are summarized in Supplementary Table 1. Several risk-factor distributions differed between groups. A smaller proportion of DR patients reported shorter weekday outdoor time compared to non-DR patients. Additionally, DR patients had significantly higher systolic blood pressure (*p* = 0.012). Three feature-selection methods were employed to identify variables associated with DR, including random forest, Lasso regression and stepwise regression. Random forest analysis (bubble plot) identified systolic blood pressure, weight, diabetes duration, cholesterol, diastolic blood pressure, weekday outdoor time, and age at diabetes diagnosis as the most influential features (Figure 2A). LASSO regression selected eight predictors: high cholesterol, sunscreen use, weight, annual household income, diabetes duration, total cholesterol, systolic blood pressure, and weekday outdoor time (Figures 2B,C). Diagnostic plots confirmed model adequacy (Figure 2D).

**FIGURE 3:**
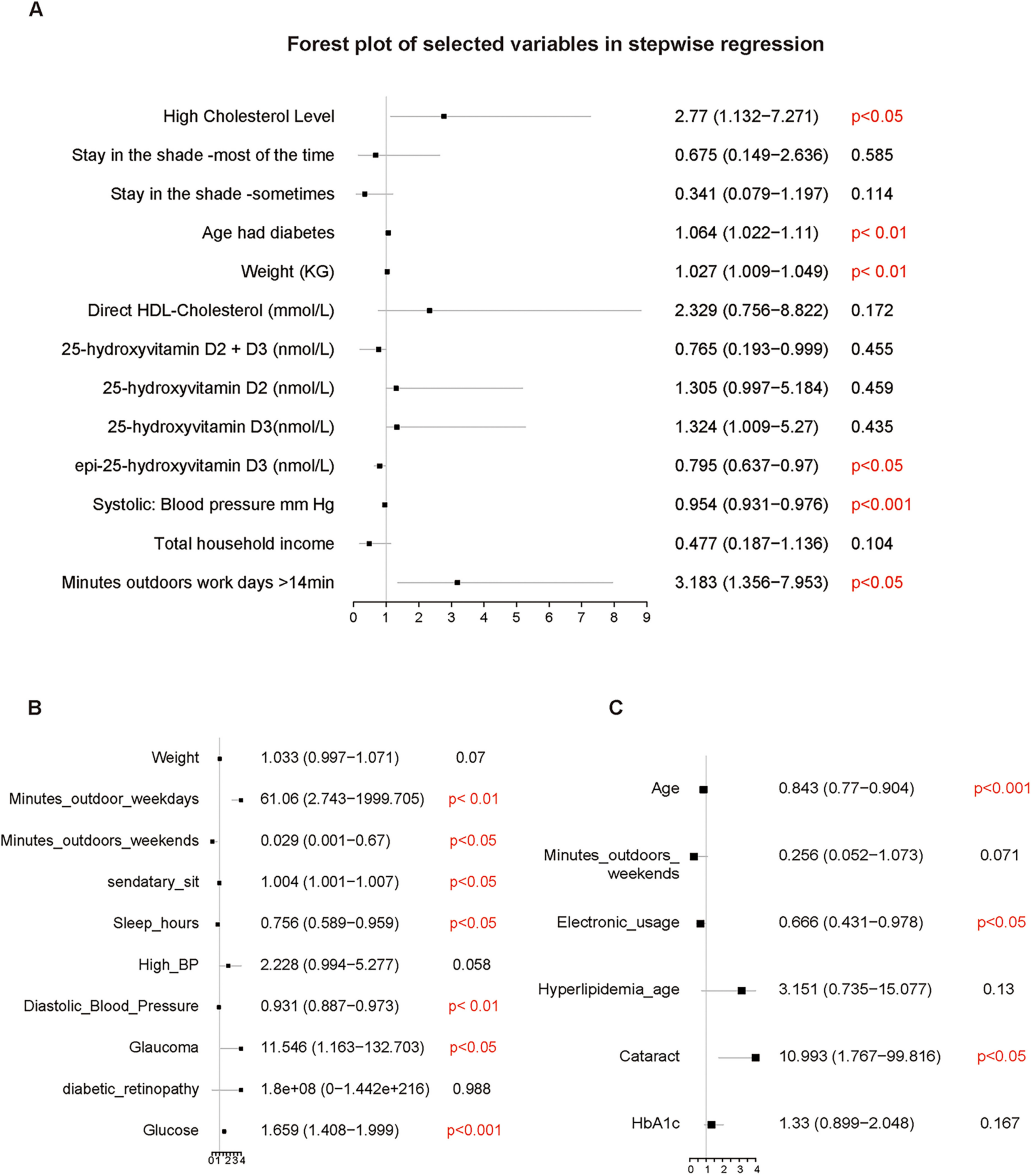
Forest plots of risk factors identified by stepwise regression models across different cohorts. **(A)** Factors associated with diabetic retinopathy in the NHANES cohort. **(B)** Factors associated with diabetes diagnosis in the self-collected clinical cohort. **(C)** Factors associated with diabetic retinopathy among diabetic patients in the self-collected cohort.

Stepwise regression selected 13 variables associated with DR (Figure 3A). Elevated cholesterol (OR = 2.77, 95% CI: 1.132–7.271), later age at diabetes diagnosis (OR = 1.064, 95% CI: 1.022–1.110), higher weight (OR = 1.027, 95% CI: 1.009–1.049), and extended weekday outdoor time > 14 minutes (OR = 3.183, 95% CI: 1.356–7.953) showed positive associations with DR. Higher epi-25-hydroxyvitamin D3 was inversely associated with DR (OR = 0.795, 95% CI: 0.637–0.970), a direction contrary to clinical expectation. Notably, systolic blood pressure was also inversely associated with DR (OR = 0.954, 95% CI: 0.931–0.976).

### Associations in the self-collected clinical dataset

Among 300 patients with ocular conditions in the clinical cohort, 84 were diagnosed with diabetes, and 34 of these had DR. Baseline characteristics of the overall cohort and the diabetic subgroup are summarized in Supplementary Table 2 and 3, respectively. Analyses were divided into two parts: first, we assessed factors associated with a diabetes diagnosis in the overall clinical cohort; second, we evaluated predictors of DR among diabetic patients, aligning with the NHANES-based strategy. For the feature selection, we applied random forest, LASSO, and bidirectional stepwise regression. Random forest algorithm identified age, cholesterol level, sleep duration, weekend outdoor time, systolic blood pressure, electronic device usage, HbA1c, weight, and glaucoma as the most influential variables (Supplementary Figure 1A). However, in multivariate logistic regression adjusted for the above features, only age remained statistically significant (Supplementary Figure 1B). LASSO regression selected four key features: age, weekend outdoor time, sleep duration, and pterygium (Supplementary Figure 1C). In the resulting model, each one-year increase in age was associated with lower odds of diabetes (OR = 0.883, 95% CI: 0.821–0.936, *p* < 0.001) (Supplementary Figure 1D). Weekend outdoor time showed a marginal, non-significant protective trend (OR < 1, *p* = 0.079).

Bidirectional stepwise regression analysis further elucidated the multivariable with diabetes diagnosis (Figure 3B). The direction of association differed by weekday versus weekend outdoor exposure: prolonged weekday exposure (> 30 min) was associated with substantially higher odds of diabetes (OR = 61.06, 95% CI: 2.74–1999.71, *p* < 0.01), whereas equivalent weekend exposure was associated with markedly lower odds (OR = 0.029, 95% CI: 0.001–0.67, *p* < 0.05). Additional factors associated with higher odds of diabetes included longer sedentary time (OR = 1.004, 95% CI: 1.001–1.007, *p* < 0.05), glaucoma (OR = 11.55, 95% CI: 1.16–132.70, p < 0.05), and elevated glucose levels. Conversely, longer sleep duration (OR = 0.756, 95% CI: 0.59–0.96, *p* < 0.05) and higher diastolic blood pressure (OR = 0.931, 95% CI: 0.887–0.973, *p* < 0.05) were associated with lower odds. For the DR-specific analysis, stepwise regression was used for better model interpretability, though results require cautious interpretation due to potential overfitting. Older age (OR = 0.843, 95% CI: 0.770–0.904, *p* < 0.001) and less electronic device usage (OR = 0.666, 95% CI: 0.431–0.978, *p* < 0.05) were associated with lower odds of DR (Figure 3C). Conversely, cataract comorbidity was strongly associated with higher odds of DR (OR = 10.993, 95% CI: 1.767–99.816, *p* < 0.05). Weekend outdoor time showed a non-significant protective trend (*p* = 0.071).

### Association between urinary phthalate metabolites and diabetic retinopathy

**FIGURE 4:**
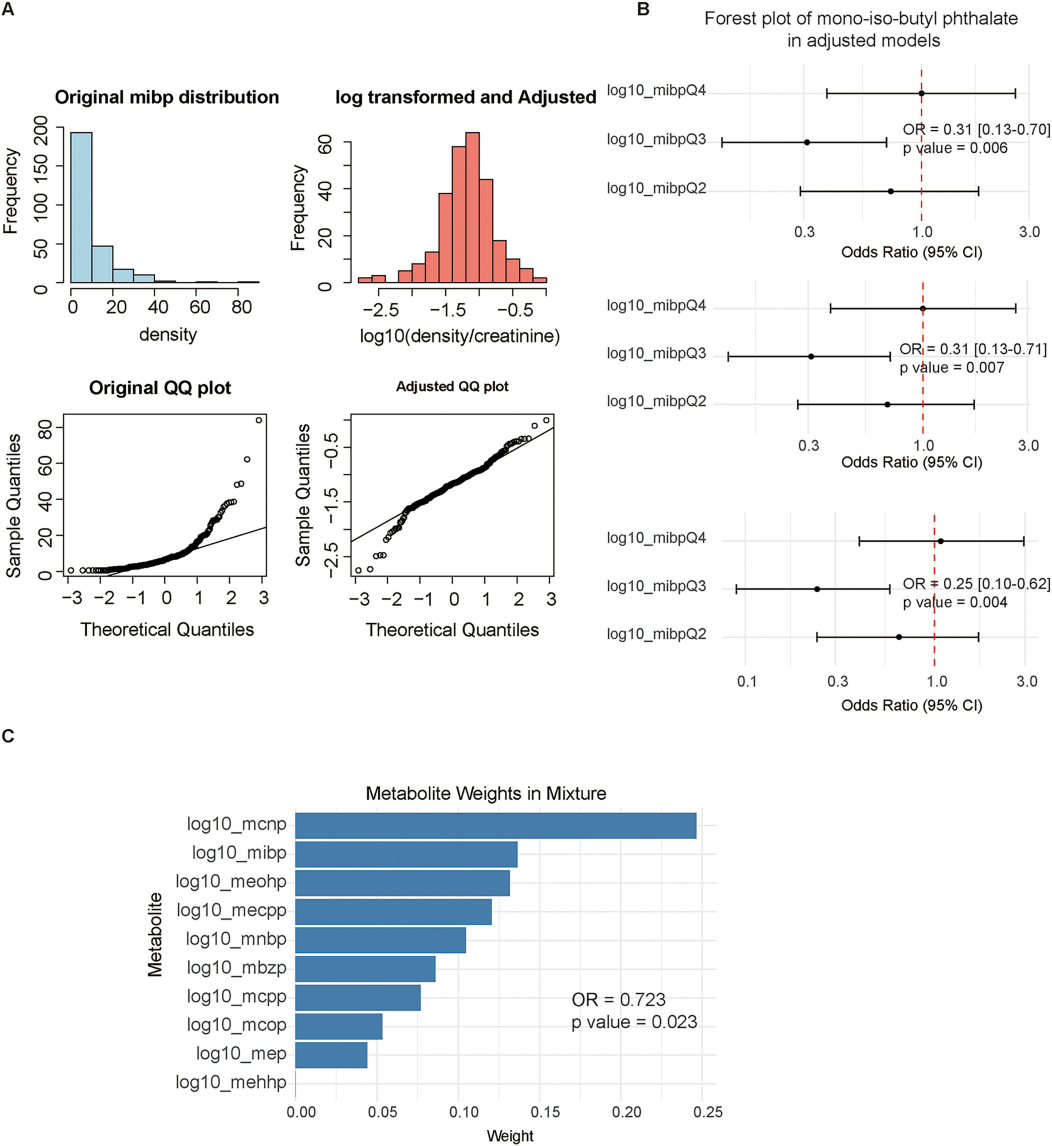
Associations between urinary phthalate metabolites and diabetic retinopathy. **(A)** Distribution of urinary phthalate metabolite concentrations. **(B)** Associations of individual phthalate metabolites with DR. **(C)** Contribution of individual metabolites to the mixture association.

Baseline characteristics of the cohort with urinary phthalate metabolites are summarized in Supplementary Table 4. The distribution of individual metabolites is presented in Supplementary Table 5. Due to the left-skewed nature of the data, metabolite concentrations were log10-transformed to approximate a normal distribution for subsequent analyses (Figure 4A and Supplementary Figures 2, 3). We employed multivariate logistic regression to examine relationships between individual phthalate metabolites and DR. As prespecified, three sequential models were constructed: Model 1 (unadjusted), Model 2 (adjusted for gender, race, and age), and Model 3 (further adjusted for education level and alcohol use). Across all models, only the third quartile (Q3) of MiBP showed a consistent inverse association with DR, with all ORs below 1 relative to Q1. This finding indicates lower odds of DR among individuals with MiBP concentrations in Q3 than among those in Q1 (Figure 4B and Supplementary Figure 4). Furthermore, we evaluated the effect of mixed phthalate exposure using weighted quantile sum (WQS) regression. The analysis revealed a significant inverse association between the overall phthalate mixture index and DR (OR = 0.723, *p* = 0.023; Figure 4C). Contribution analysis identified MCNP as the largest contributor to this association, followed by MiBP, MEOHP, and MECPP.

### Identification and functional analysis of common target genes

**FIGURE 5:**
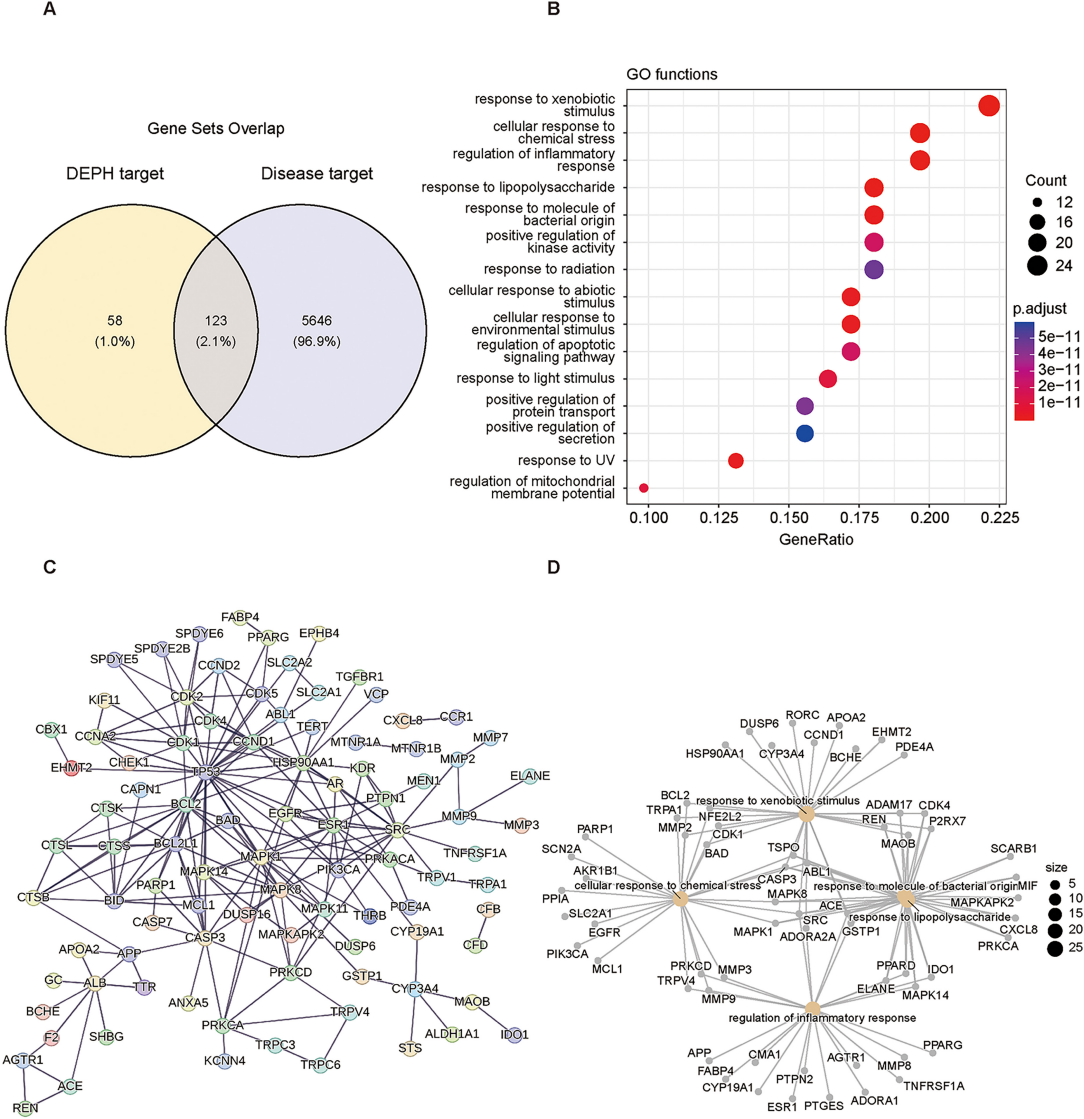
Identification and functional analysis of common targets between DEHP and diabetic retinopathy. **(A)** Venn diagram of the candidate targets. **(B)** Gene Ontology (GO) biological process enrichment of common targets. **(C)** Protein-protein interaction (PPI) network. **(D)** Functional module network of key genes.

Using “DEHP” as the query term, we identified 181 unique potential target genes from ChEMBL, SwissTargetPrediction, and PharmMapper databases (contributing 11, 119, and 74 genes, respectively). In parallel, 5,769 DR-associated genes were retrieved from TTD, DisGeNET, and GeneCards (contributing 6, 2, and 5,768 genes, respectively). Intersection analysis revealed 123 overlapping genes between the DEHP target set and the DR gene set (Figure 5A), which were defined as common targets for subsequent analysis. GO enrichment analysis demonstrated that the 123 common targets were significantly enriched in biological processes related to cellular defense and stress response. Prominent terms included “response to xenobiotic stimulus,” “cellular response to chemical stress,” and “regulation of inflammatory response.” Several environment-responsive terms were also enriched, such as “response to radiation,” “response to UV,” and “response to light stimulus” (Figure 5B). A PPI network was constructed using the STRING database (confidence score > 0.9; first shell interactors ≤ 5) and visualized in Cytoscape (Figure 5C). Topological analysis indicated several highly connected hub genes, including CDK1, CDK2, and CDK3, suggesting that cell-cycle-related regulatory nodes may play central roles within the common target network. Genes contributing to the most significantly enriched GO terms are displayed in Figure 5D.

**FIGURE 6:**
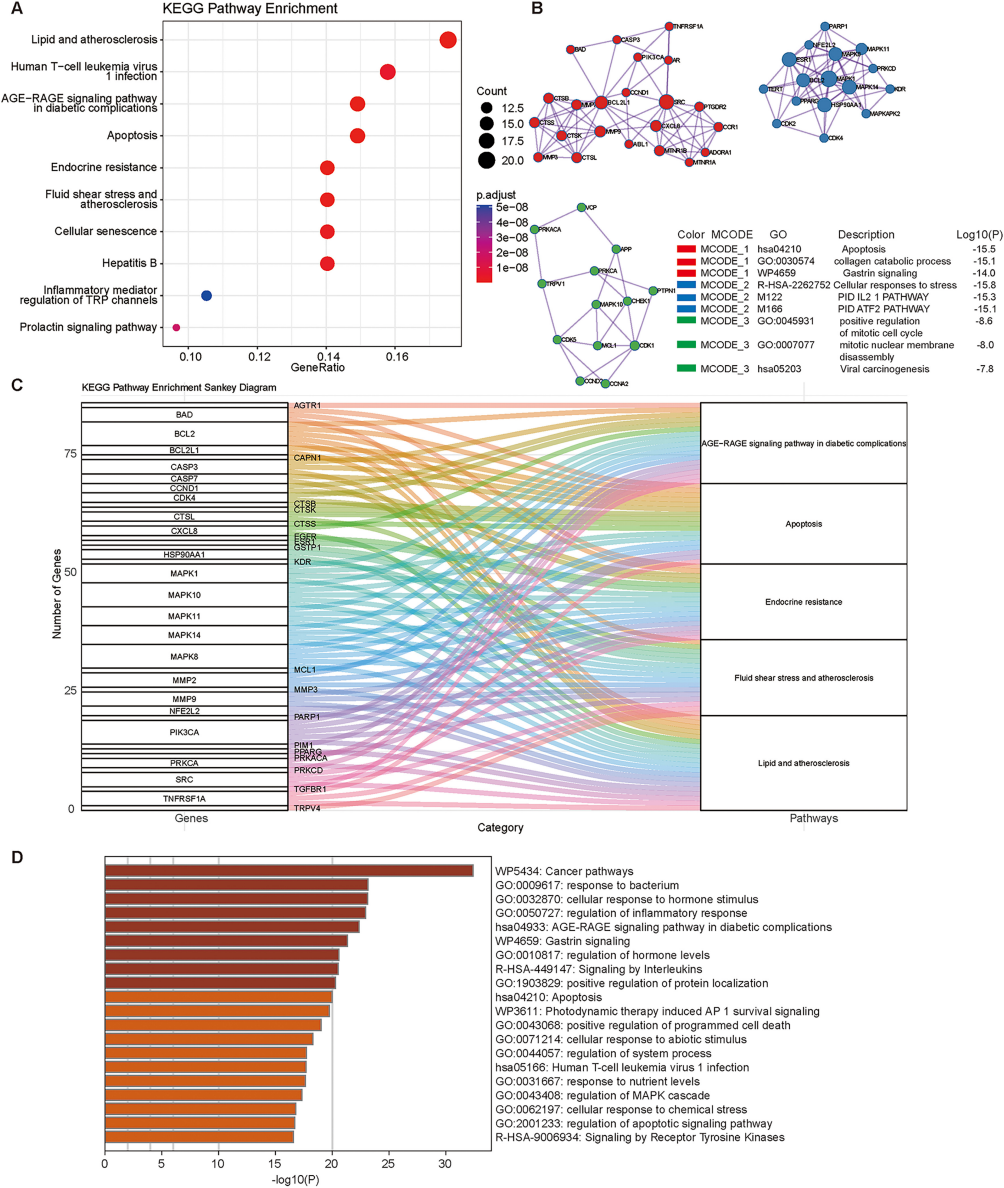
Integrated functional enrichment and network analysis of common targets. **(A)** KEGG pathway enrichment analysis. **(B)** Functional module analysis. **(C)** Gene-pathway association network. **(D)** Comprehensive functional heatmap.

KEGG pathway analysis further revealed significant enrichments in several key pathways, most notably “Lipid and atherosclerosis,” “AGE-RAGE signaling pathway in diabetic complications,” and “Apoptosis.” Additional relevant pathways included “Endocrine resistance,” “Cellular senescence,” and “Inflammatory mediator regulation of TRP channels.” All enriched pathways were statistically significant (adjusted p-value range: ∼1 × 10^−8^ to 5 × 10^−8^), with GeneRatio values ranging from 0.10 to 0.16, implicating the common targets in critical processes spanning metabolic disease, diabetic complications, and cellular regulation (Figure 6A). Collectively, these findings suggest a potential link between phthalate exposure and DR-related biology. Network toxicology identified a set of shared genes connecting DEHP-associated targets to DR, with enrichment in established DR-relevant pathways, including AGE–RAGE signaling, apoptosis, and cellular senescence. Given the central role of AGE–RAGE signaling in hyperglycemia-driven vascular injury, these results are consistent with a plausible model in which environmental chemical exposure may interact with metabolic stress to potentiate retinal vascular dysfunction.

MCODE clustering identified three highly interconnected functional modules within the PPI network (Figure 6B). MCODE_1 was significantly enriched in “Apoptosis,” “collagen catabolic process,” and “Gastrin signaling.” MCODE_2 was associated with “Cellular responses to stress,” “PID IL2 pathway,” and “PID ATF2 pathway.” MCODE_3 was linked to the “positive regulation of the mitotic cell cycle,” “mitotic nuclear membrane disassembly,” and “Viral carcinogenesis.” These modules collectively highlight three major functional themes within the shared DEHP-DR network: cell death/remodeling, stress response/signaling, and cell cycle regulation/viral pathogenesis.

### Integrated network visualization and topological analysis

**FIGURE 7:**
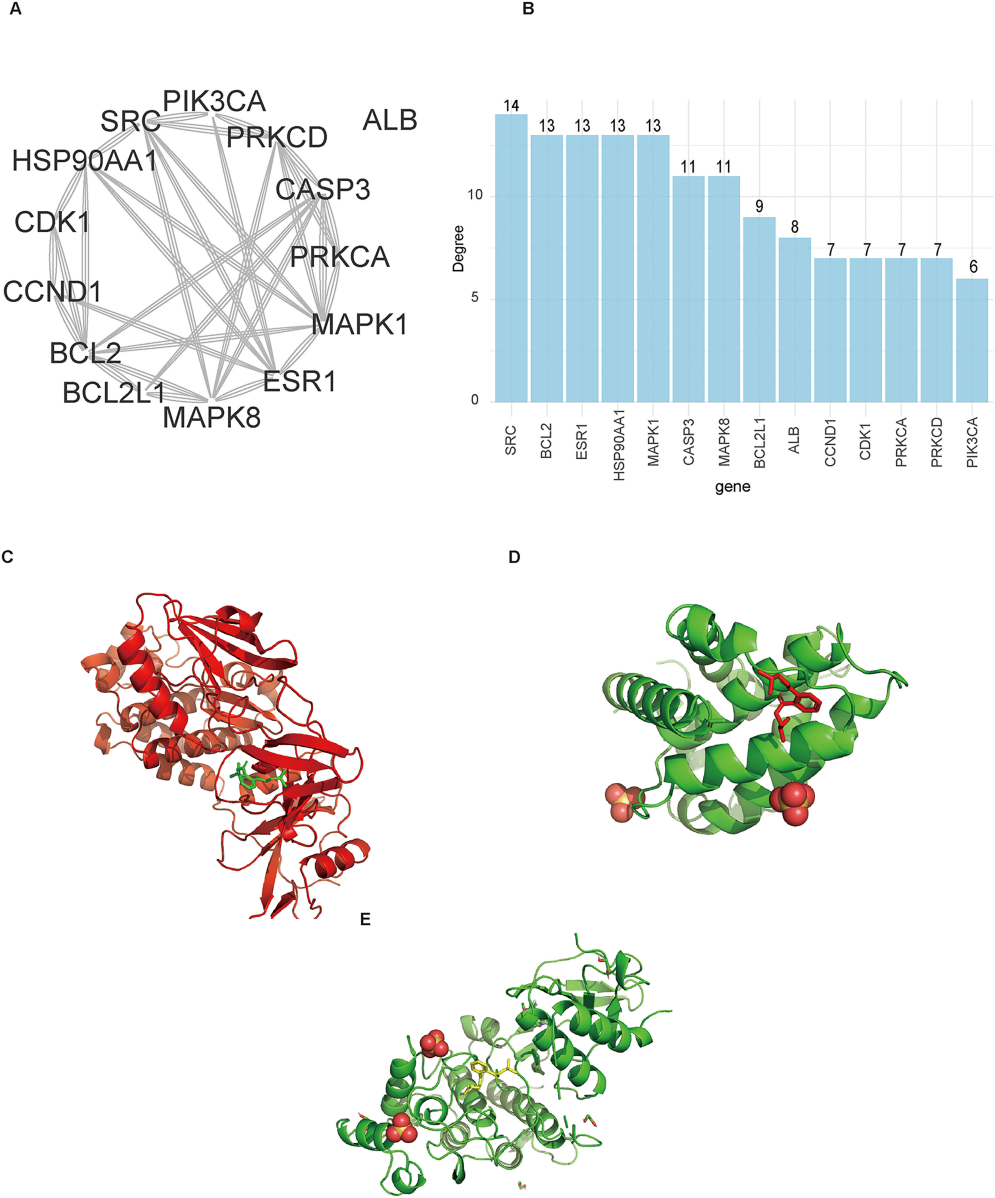
Topological analysis of core targets and molecular docking validation. **(A)** Identification of hub genes in the protein-protein interaction network. **(B)** Degree centrality ranking of core targets. **(C–E)** Predicted binding modes of DEHP with core targets.

To depict the relationships between core genes and significantly enriched pathways, we generated a Sankey diagram summarizing gene–pathway connectivity (Figure 6C). Several genes—notably PIK3CA, MAPK1, MAPK14, CASP3, and BCL2—were connected to multiple pathways, including “Lipid and atherosclerosis,” “AGE-RAGE signaling pathway in diabetic complications,” and “Apoptosis.” This pattern suggests potential pleiotropic roles for these core genes as the intersection of metabolic disease, diabetic complications, and programed cell death. A functional heatmap further consolidated pathway-level signals, consistent with the involvement of stress, inflammatory, and cell death-related processes identified in the GO analyses (Figure 6D). Moreover, the heatmap expanded the functional context by incorporating disease-specific pathways from both KEGG and Reactome databases, showing links to diabetes-, cancer-, and infection-related pathways. Topological analysis of the common-target PPI network was performed using the CytoNCA plugin in Cytoscape. Genes were ranked by their degree centrality, and the resulting core targets (degree ≥ 6) were visualized in a circular layout (Figure 7A). The corresponding degree values of these high-connectivity genes are displayed in a bar plot (Figure 7B), identifying the most highly connected nodes within the DEHP-DR interaction network.

### Molecular docking validation

Molecular docking was performed to evaluate the binding affinity between DEHP and the core targets SRC, BCL2, and MAPK1. The proteins ESR1 and HSP90AA1 were excluded from this analysis due to the lack of available three-dimensional structures suitable for docking. DEHP exhibited favorable predicted binding energies with all three targets: SRC (−6.0 kcal/mol), BCL2 (−5.53 kcal/mol), and MAPK1 (−5.04 kcal/mol). All docking scores were below −5.0 kcal/mol, suggesting stable binding interactions between DEHP and these candidate targets (Figures 7C–E).

## Discussion

Evidence indicates that multiple risk factors are associated with mortality and life expectancy among individuals with diabetes (28–30). This study utilized nationally representative NHANES 2017–2018 data to identify risk factors associated with DR, and further evaluated these associations in an independent, self-collected clinical cohort. In the NHANES analysis, six variables were retained as significant correlates of DR: high cholesterol level, age at diabetes diagnosis, weight, epi-25-hydroxyvitamin D3 level, systolic blood pressure, and weekday outdoor time. Among these, epi-25-hydroxyvitamin D3 showed an inverse association with DR, with higher levels associated with lower odds of DR. This represents a novel observation that has not been emphasized in prior reports, while remaining broadly consistent with literature linking adequate vitamin D status to reduce DR risk (31–33). Conversely, increased weekday outdoor time (> 14 min between 09:00 and 17:00) was associated with increased odds of DR. This pattern is in line with previous findings reporting a higher prevalence of DR among individuals with prolonged daily sunlight exposure (34), suggesting that environmental exposure (e.g., light/UV) or correlated behavioral factors related to daytime outdoor activity may play a role in DR development and progression.

In the self-collected clinical cohort, we identified seven variables significantly associated with diabetes. A notable finding was the opposing direction of association for outdoor exposure by day type: spending more than 30 min outdoors on weekdays was associated with an increased risk of diabetes, whereas equivalent outdoor exposure on weekends was associated with lower odds. This discrepancy may reflect differences in the context and intensity of outdoor activities. Weekday exposure likely occurs in occupational settings, potentially involving prolonged sunlight exposure, physical strain, and psychosocial stress, while weekend outdoor time may be more discretionary and recreational and physiologically beneficial. Functional enrichment analysis identified enriched GO terms, including “regulation of apoptotic signaling pathway,” “positive regulation of kinase activity,” and “positive regulation of protein transport,” suggesting the involvement of stress response and inflammatory processes. Additionally, prolonged sedentary behavior was associated with a higher diabetes risk, consistent with previous studies linking sedentary behavior to adverse metabolic outcomes (35, 36).

Among the diabetic subgroup, electronic device usage was inversed associated with DR, suggesting that reduced screen exposure may be linked to reduced DR risk and potentially slower progression. Several findings from the clinical cohort were inconsistent with established knowledge. For instance, older age was associated with lower odds of diabetes, which contrasts with the well-established positive relationship between age and diabetes. This discrepancy is likely attributable to selection bias inherent in a hospital-based ophthalmology cohort, in which younger patients may have been admitted for conditions associated with a higher prevalence of diabetes, such as aggressive diabetic retinopathy. Therefore, these associations should be interpreted with caution and should not be generalized to the broader diabetic population.

Our analyses suggested an inverse relationship between urinary phthalate mixture concentrations and DR. This finding differs from previous reports in which higher levels of endocrine-disrupting chemical (EDC) were associated with increased all-cause mortality among individuals with DR (21), highlighting the complexity of exposure-outcome relationships and the potential for outcome-specific effects. Given the limited literature directly evaluating phthalates in relation to DR, our results suggest that further investigation is warranted into environmental pollutants and diabetic microvascular complications (37). In metabolite-specific models, generalized linear model (GLM) analysis identified MiBP as independently associated with DR. Consistently, weighted quantile sum (WQS) regression showed a significant negative correlation between the mixed phthalate index and DR, with MCNP, MiBP, and MEOHP contributing most strongly to the mixture signal. Although this inverse association is unexpected from a biological perspective and requires replication, MiBP has been linked to renal impairment, periodontitis, and aging-related phenotypes (38–41), its potential involvement in diabetic complications remains defined and warrants deeper mechanistic investigation.

Accumulating evidence links phthalate exposure to oxidative stress and inflammation. One study in pregnant women reported that most urinary phthalate metabolites were associated with increased 8-iso-PGF2α, a biomarker reflecting both oxidative stress and inflammation (42). Another cross-sectional study in adults found that co-exposure to phthalate was associated with elevated oxidative stress and identified monoisobutyl phthalate (MiBP) as the metabolite with the strongest contribution to this effect (43). Experimental evidence from animal models further demonstrated that dibutyl phthalate induces cardiac oxidative stress and inflammation through the Nrf2/NF-κB signaling pathway (41). A systematic review likewise concluded that phthalates may influence metabolic disorders through mechanisms involving oxidative stress, inflammation, and PPAR activation (44). In parallel, substantial evidence supports key roles for oxidative stress and inflammation in diabetic retinopathy. The deubiquitylase OTUD3 protects retinal function by deubiquitylating PPARγ, while a PPARγ agonist reduces ROS levels, inhibits excessive cell migration, and decreases apoptosis in retinal pigment epithelial cells under inflammatory conditions (45). Inhibition of Cullin3 neddylation activates Nrf2 signaling, which suppresses ROS-induced oxidative stress and inflammation and ameliorates blood-retinal barrier disruption in diabetic models (46). In addition, inflammation, endoplasmic reticulum stress, oxidative stress, and autophagy have all been recognized as major contributors to DR pathogenesis, and melatonin has been reported to alleviate retinal injury by modulating these processes (47).

Network toxicology identified 123 overlapping genes connecting DEHP exposure to DR, with functional enrichment in DR-related pathways such as AGE-RAGE signaling, apoptosis, and cellular senescence. These results raise the possibility that phthalate exposure may exacerbate core pathological processes of DR. The central involvement of AGE-RAGE pathway, a well-established axis mediating hyperglycemia-induced vascular damage, suggests the possibility that metabolic stress and environmental chemical exposure may act synergistically to aggravate retinal damage in diabetes. Molecular docking predicted stable interactions between the DEHP metabolite MiBP and three core targets—SRC, BCL2, and MAPK1—all of which embedded in the identified KEGG pathways. SRC has been proposed as a potential target for DR treatment in studies of traditional medicine, alongside its involvement in DEHP-related prostate cancer and allergic diseases (48–50). Similarly, BCL2 and MAPK1 have been reported to exhibit favorable binding with DEHP in multiple disease contexts (39, 51–53), suggesting their potential toxicological relevance. Together, these *in silico* findings support the hypothesis that MiBP may influence DR-relevant biology through multiple parallel mechanisms, potentially altering apoptotic thresholds, enhancing inflammatory signaling, and amplifying cellular stress responses, although experimental validation is still required.

This study has several limitations. First, inconsistencies in variable availability between NHANES and the validation datasets, largely due to practical constraints in clinical data collection, limited the direct comparability of findings across datasets. Second, the sample size was constrained by the number of confirmed DR cases in both cohorts, which may have reduced statistical power, particularly in subgroup analyses. Third, the cross-sectional design of NHANES precludes causal inference; therefore, all reported associations should be interpreted as correlational rather than causal. Fourth, the use of stepwise regression, which was primarily intended to improve model interpretability, carries a risk of overfitting and may yield unstable coefficient estimates. As such, the identified variables require validation in independent datasets. In addition, some inverse associations, such as those involving blood pressure and phthalates, are biologically unexpected and may reflect residual confounding or chance. Finally, the *in silico* molecular findings remain hypothesis-generating and require experimental validation. Larger studies with harmonized variable collection and standardized DR ascertainment are needed to validate these associations and strengthen causal inference.

## Conclusion

This study corroborates established DR-associated factors and identifies additional associations, including weekday outdoor time and specific urinary phthalate metabolites. By integrating population-based analyses with network toxicology, our findings suggest that phthalate exposure may be associated with DR pathogenesis through core pathways such as AGE–RAGE signaling and apoptosis. Molecular docking predicted stable binding of MiBP to key targets (SRC, BCL2, and MAPK1), offering mechanistic hypotheses for future validation. Collectively, these results preliminarily highlight the potential importance of environmental exposures in DR risk stratification and pathobiology. The inverse association observed for electronic device use also raises the possibility that modifiable behaviors may contribute to DR prevention, although this relationship requires confirmation. Future work in larger prospective cohorts, coupled with experimental validation, will be essential to clarify these associations and delineate the underlying mechanisms, thereby informing more comprehensive strategies for DR prevention and management.

## Data Availability

The original contributions presented in this study are included in the article/Supplementary material, further inquiries can be directed to the corresponding authors.
