# Supplementary material for: From risk factors to molecular targets: clinical associations and molecular docking insights into phthalate-associated diabetic retinopathy
Source: Front Med (Lausanne). 2026 May 13;13:1792532. doi: 10.3389/fmed.2026.1792532 (PMC13212054; doi:10.3389/fmed.2026.1792532)
Supplement: Supplementary file 5 [file Table_1.docx]

Supplementary Table 1. Baseline Characteristics of the Study Population- NHANES.

| **Characteristic** | **Retinopathy**, N = 42^1^ | **No Retinopathy**, N = 166^1^ | **p-value**^2^ |
| --- | --- | --- | --- |
| Male | 22 (52%) | 83 (50%) |  |
| Female | 20 (48%) | 83 (50%) |  |
| **Age (years)** | 49 (7) | 48 (8) | 0.7 |
| **Ethnicity1** |  |  | 0.8 |
| Mexican American | 4 (9.5%) | 26 (16%) |  |
| Other Hispanic | 3 (7.1%) | 12 (7.2%) |  |
| Non-Hispanic White | 15 (36%) | 47 (28%) |  |
| Non-Hispanic Black | 9 (21%) | 35 (21%) |  |
| Other Race - Including Multi-Racial | 11 (26%) | 46 (28%) |  |
| **Ethnicity2** |  |  | >0.9 |
| Mexican American | 4 (9.5%) | 26 (16%) |  |
| Other Hispanic | 3 (7.1%) | 12 (7.2%) |  |
| Non-Hispanic White | 15 (36%) | 47 (28%) |  |
| Non-Hispanic Black | 9 (21%) | 35 (21%) |  |
| Non-Hispanic Asian | 8 (19%) | 31 (19%) |  |
| Other Race - Including Multi-Racial | 3 (7.1%) | 15 (9.0%) |  |
| **Education** |  |  | 0.7 |
| Less than 9th grade | 2 (4.8%) | 16 (9.6%) |  |
| 9-11th grade (Includes 12th grade with no diploma) | 7 (17%) | 19 (11%) |  |
| High school graduate/GED or equivalent | 11 (26%) | 34 (20%) |  |
| Some college or AA degree | 14 (33%) | 63 (38%) |  |
| College graduate or above | 8 (19%) | 34 (20%) |  |
| **High Blood Pressure** |  |  | 0.4 |
| Yes | 25 (60%) | 86 (52%) |  |
| No | 17 (40%) | 80 (48%) |  |
| **High Cholesterol Level** |  |  | 0.032 |
| Yes | 28 (67%) | 80 (48%) |  |
| No | 14 (33%) | 86 (52%) |  |
| **Skin reaction to sun** |  |  | >0.9 |
| Get a severe sunburn with blisters | 1 (2.4%) | 5 (3.0%) |  |
| A severe sunburn for a few days with peeling | 3 (7.1%) | 17 (10%) |  |
| Mildly burned with some tanning | 12 (29%) | 40 (24%) |  |
| Turning darker without a sunburn | 5 (12%) | 27 (16%) |  |
| Nothing would happen in half an hour | 21 (50%) | 77 (46%) |  |
| **Stay in the shade** |  |  | 0.4 |
| Always | 4 (9.5%) | 27 (16%) |  |
| Most of the time | 12 (29%) | 59 (36%) |  |
| Sometimes | 19 (45%) | 61 (37%) |  |
| Rarely | 7 (17%) | 19 (11%) |  |
| **Use sunscreen** |  |  | 0.5 |
| Always | 4 (9.5%) | 17 (10%) |  |
| Most of the time | 2 (4.8%) | 15 (9.0%) |  |
| Sometimes | 5 (12%) | 34 (20%) |  |
| Rarely | 9 (21%) | 38 (23%) |  |
| Never | 22 (52%) | 62 (37%) |  |
| **Sunburn** |  |  | 0.9 |
| Yes | 14 (33%) | 53 (32%) |  |
| No | 28 (67%) | 113 (68%) |  |
| **Minutes outdoors work day** | 77 (124) | 77 (107) | 0.4 |
| **Minutes outdoors weekend** | 130 (103) | 138 (123) | >0.9 |
| Diabetes diagnosed age | 37 (12) | 40 (10) | 0.12 |
| **Minutes sedentary activity** | 341 (244) | 365 (215) | 0.3 |
| **Trouble sleeping** |  |  | 0.5 |
| Yes | 22 (52%) | 73 (44%) |  |
| No | 20 (48%) | 92 (55%) |  |
| Don’t know | 0 (0%) | 1 (0.6%) |  |
| **60 second pulse** | 78 (11) | 79 (13) | 0.4 |
| **Weight (KG)** | 89 (18) | 99 (30) | 0.083 |
| **Body Mass Index** | 34 (10) | 35 (10) | 0.3 |
| **Direct HDL-Cholesterol (mmol/L)** | 1.19 (0.32) | 1.21 (0.40) | 0.6 |
| **Total cholesterol (mmol/L)** | 5.20 (1.79) | 4.74 (1.11) | 0.3 |
| **25OHD2+25OHD3 (nmol/L)** | 69 (34) | 69 (32) | 0.9 |
| **25OHD2 (nmol/L)** | 9 (22) | 8 (19) | 0.3 |
| **25OHD3 (nmol/L)** | 60 (35) | 62 (34) | 0.6 |
| **epi-25OHD3 (nmol/L)** | 4.12 (3.56) | 3.78 (2.71) | 0.9 |
| **Diastolic Blood pressure mmHg** | 75 (11) | 76 (10) | 0.8 |
| **Systolic Blood pressure mmHg** | 137 (26) | 125 (16) | 0.012 |
| **Glycohemoglobin (%)** | 8.17 (2.01) | 7.59 (1.92) | 0.066 |
| **Annual Household Income** |  |  | 0.070 |
| high | 12 (29%) | 73 (44%) |  |
| low | 30 (71%) | 93 (56%) |  |
| **Cholesterol, total (mg/dL)** | 200 (70) | 185 (44) | 0.4 |
| **Triglycerides (mg/dL)** | 183 (105) | 196 (176) | >0.9 |
| **Minutes outdoors workdays** |  |  | 0.2 |
| 0 (<= 14 minutes) | 22 (52%) | 68 (41%) |  |
| 1 | 20 (48%) | 98 (59%) |  |
| ^1^n (%); Mean (SD) | | | |
| ^2^Pearson's Chi-squared test; Wilcoxon rank sum test; Fisher's exact test | | | |
