# Supplementary material for: From risk factors to molecular targets: clinical associations and molecular docking insights into phthalate-associated diabetic retinopathy
Source: Front Med (Lausanne). 2026 May 13;13:1792532. doi: 10.3389/fmed.2026.1792532 (PMC13212054; doi:10.3389/fmed.2026.1792532)
Supplement: Supplementary file 6 [file Table_2.docx]

Supplementary Table 2. Baseline Characteristics of the Study Population- clinical cohort.

| **Characteristics** | **Level** | **No diabetes** | **Diabetes** | **P value** |
| --- | --- | --- | --- | --- |
|  |  | 216 | 84 |  |
| **Gender** (%) | Female | 122 (56.5) | 39 (46.4) | 0.15 |
|  | Male | 94 (43.5) | 45 (53.6) | |
| **Shade in sun** (%) | No | 158 (73.1) | 63 (75.0) | 0.856 |
|  | Yes | 58 (26.9) | 21 (25.0) | |
| **Intensive work** (%) | No | 183 (84.7) | 71 (84.5) | 1 |
|  | Yes | 33 (15.3) | 13 (15.5) | |
| **Trouble sleeping** (%) | No | 204 (94.4) | 76 (90.5) | 0.327 |
|  | Yes | 12 (5.6) | 8 (9.5) | |
| **Smoking history** (%) | No | 184 (85.2) | 66 (78.6) | 0.227 |
|  | Yes | 32 (14.8) | 18 (21.4) | |
| **High Blood Pressure** (%) | No | 114 (52.8) | 29 (34.5) | 0.007 |
|  | Yes | 102 (47.2) | 55 (65.5) | |
| **Hyperlipidemia age** (%) | No | 195 (90.3) | 64 (76.2) | 0.003 |
|  | Yes | 21 (9.7) | 20 (23.8) | |
| **Diabetic symptoms** (%) | No | 216 (100.0) | 35 (41.7) | <0.001 |
|  | Yes | 0 (0.0) | 49 (58.3) | |
| **Glaucoma** (%) | No | 214 (99.1) | 79 (94.0) | 0.03 |
|  | Yes | 2 (0.9) | 5 (6.0) | |
| **Macular_degeneration** (%) | No | 210 (97.2) | 83 (98.8) | 0.695 |
|  | Yes | 6 (2.8) | 1 (1.2) | |
| **Cataract** (%) | No | 33 (15.3) | 13 (15.5) | 1 |
|  | Yes | 183 (84.7) | 71 (84.5) | |
| **Diabetic retinopathy** (%) | No | 216 (100.0) | 50 (59.5) | <0.001 |
|  | Yes | 0 (0.0) | 34 (40.5) | |
| **Pterygium** (%) | No | 178 (82.4) | 74 (88.1) | 0.302 |
|  | Yes | 38 (17.6) | 10 (11.9) | |
| **Age** (mean (SD)) | | 68.47 (10.44) | 64.73 (11.29) | 0.007 |
| **Weight** (mean (SD)) | | 60.69 (11.77) | 64.92 (11.52) | 0.005 |
| **Height** (mean (SD)) | | 158.81 (15.11) | 162.75 (8.04) | 0.024 |
| **Minutes outdoor weekdays** (%) | <= 30 min | 49 (22.7) | 18 (21.4) | 0.936 |
|  | > 30 min | 167 (77.3) | 66 (78.6) | |
| **Minutes outdoors weekends** (%) | <= 30 min | 41 (19.0) | 23 (27.4) | 0.151 |
|  | > 30 min | 175 (81.0) | 61 (72.6) | |
| **Drink frequency year**  (mean (SD)) | | 37.61 (102.45) | 33.82 (91.50) | 0.768 |
| **Intensive workout hours**  (mean (SD)) | | 0.56 (1.60) | 0.91 (1.93) | 0.105 |
| **Electronic usage**  (mean (SD)) | | 2.26 (1.72) | 2.39 (1.70) | 0.57 |
| **Sedentary sit**  (mean (SD)) | | 154.94 (114.98) | 191.43 (126.08) | 0.017 |
| **Sleep hours**  (mean (SD)) | | 6.71 (1.68) | 6.51 (1.75) | 0.356 |
| **Systolic Blood Pressure**  (mean (SD)) | | 139.88 (16.23) | 137.29 (16.19) | 0.214 |
| **Diastolic Blood Pressure**  (mean (SD)) | | 77.80 (43.68) | 73.88 (8.47) | 0.415 |
| **Triglyceride** (mean (SD)) | | 1.83 (1.08) | 2.00 (1.09) | 0.232 |
| **Cholesterol** (mean (SD)) | | 5.06 (1.16) | 4.68 (1.19) | 0.013 |
| **Glucose** (mean (SD)) | | 5.48 (1.73) | 8.60 (3.87) | <0.001 |
| **HbA1c** (mean (SD)) | | 23.12 (252.51) | 7.68 (1.60) | 0.576 |
