# Supplementary material for: From risk factors to molecular targets: clinical associations and molecular docking insights into phthalate-associated diabetic retinopathy
Source: Front Med (Lausanne). 2026 May 13;13:1792532. doi: 10.3389/fmed.2026.1792532 (PMC13212054; doi:10.3389/fmed.2026.1792532)
Supplement: Supplementary file 7 [file Table_3.docx]

Supplementary Table 3. Baseline Characteristics of the Study Population_clinical_DR.

| **Characteristic** | **0**, N = 50^1^ | **1**, N = 34^1^ | **p-value**^2^ |
| --- | --- | --- | --- |
| **Age** | 70 (10) | 57 (9) | <0.001 |
| **Gender** |  |  | 0.092 |
| 0 | 27 (54%) | 12 (35%) |  |
| 1 | 23 (46%) | 22 (65%) |  |
| **Weight** | 64 (12) | 66 (11) | 0.5 |
| **Height** | 162 (9) | 164 (7) | 0.2 |
| **Minutes_outdoor_weekdays** |  |  | 0.044 |
| 0 | 7 (14%) | 11 (32%) |  |
| 1 | 43 (86%) | 23 (68%) |  |
| **Minutes_outdoors_weekends** |  |  | 0.005 |
| 0 | 8 (16%) | 15 (44%) |  |
| 1 | 42 (84%) | 19 (56%) |  |
| **Shade_in_sun** |  |  | 0.2 |
| 0 | 40 (80%) | 23 (68%) |  |
| 1 | 10 (20%) | 11 (32%) |  |
| **Electronic_usage** |  |  | 0.10 |
| 0 | 8 (16%) | 2 (5.9%) |  |
| 0.5 | 2 (4.0%) | 3 (8.8%) |  |
| 1 | 4 (8.0%) | 9 (26%) |  |
| 2 | 12 (24%) | 11 (32%) |  |
| 3 | 8 (16%) | 2 (5.9%) |  |
| 4 | 5 (10%) | 1 (2.9%) |  |
| 5 | 11 (22%) | 6 (18%) |  |
| **Intensive_work** |  |  | 0.9 |
| 0 | 42 (84%) | 29 (85%) |  |
| 1 | 8 (16%) | 5 (15%) |  |
| **sendatary_sit** | 200 (127) | 179 (125) | 0.3 |
| **Sleep_hours** | 6.12 (1.75) | 7.07 (1.61) | 0.012 |
| **trouble_sleeping** |  |  | >0.9 |
| 0 | 45 (90%) | 31 (91%) |  |
| 1 | 5 (10%) | 3 (8.8%) |  |
| **smokin_history** |  |  | 0.9 |
| 0 | 39 (78%) | 27 (79%) |  |
| 1 | 11 (22%) | 7 (21%) |  |
| **High_BP** |  |  | >0.9 |
| 0 | 17 (34%) | 12 (35%) |  |
| 1 | 33 (66%) | 22 (65%) |  |
| **Systolic_Blood_Pressure** | 139 (16) | 135 (16) | 0.2 |
| **Diastolic_Blood_Pressure** | 73 (9) | 76 (8) | 0.072 |
| **Hyperlipidemia_age** |  |  | 0.13 |
| 0 | 41 (82%) | 23 (68%) |  |
| 1 | 9 (18%) | 11 (32%) |  |
| **Triglyceride** | 1.98 (1.14) | 2.04 (1.03) | 0.6 |
| **Cholesterol** | 4.74 (1.13) | 4.60 (1.29) | 0.3 |
| **Glaucoma** |  |  | >0.9 |
| 0 | 47 (94%) | 32 (94%) |  |
| 1 | 3 (6.0%) | 2 (5.9%) |  |
| **Macular_degeneration** |  |  | >0.9 |
| 0 | 49 (98%) | 34 (100%) |  |
| 1 | 1 (2.0%) | 0 (0%) |  |
| **Cataract** |  |  | 0.9 |
| 0 | 8 (16%) | 5 (15%) |  |
| 1 | 42 (84%) | 29 (85%) |  |
| **pterygium** |  |  | 0.044 |
| 0 | 41 (82%) | 33 (97%) |  |
| 1 | 9 (18%) | 1 (2.9%) |  |
| **Glucose** | 8.6 (4.1) | 8.7 (3.5) | 0.7 |
| **HbA1c** | 7.54 (1.65) | 7.90 (1.52) | 0.2 |
| ^1^Mean (SD); n (%) | | | |
| ^2^Wilcoxon rank sum test; Pearson's Chi-squared test; Fisher's exact test | | | |
