# Supplementary material for: From risk factors to molecular targets: clinical associations and molecular docking insights into phthalate-associated diabetic retinopathy
Source: Front Med (Lausanne). 2026 May 13;13:1792532. doi: 10.3389/fmed.2026.1792532 (PMC13212054; doi:10.3389/fmed.2026.1792532)
Supplement: Supplementary file 8 [file Table_4.docx]

Supplementary Table 4. Baseline Characteristics of the Study Population- Phthalate metabolites.

| **Characteristics** | **Level** | **Retinopathy** | **No Retinopathy** | **P value** |  |  |
| --- | --- | --- | --- | --- | --- | --- |
|  |  | 57 | 214 |  |  |  |
| **Gender** (%) | Male | 26 (45.61) | 112 (52.34) | 0.4514 |  |  |
|  | Female | 31 (54.39) | 102 (47.66) |  |  |  |
| **Age** (mean (SD)) |  | 66.193 (10.831) | 63.224 (14.006) | 0.1386 |  |  |
| **Ethnicity1** (%) | Mexican American | 6 (10.53) | 29 (13.55) | 0.9552 |  |  |
|  | Other Hispanic | 5 (8.77) | 22 (10.28) |  |  |  |
|  | Non-Hispanic White | 21 (36.84) | 76 (35.51) |  |  |  |
|  | Non-Hispanic Black | 15 (26.32) | 49 (22.90) |  |  |  |
|  | Other Race - Including Multi-Racial | 10 (17.54) | 38 (17.76) |  |  |  |
| **Ethnicity3** (%) | Mexican American | 6 (10.53) | 29 (13.55) | 0.5873 |  |  |
|  | Other Hispanic | 5 (8.77) | 22 (10.28) |  |  |  |
|  | Non-Hispanic White | 21 (36.84) | 76 (35.51) |  |  |  |
|  | Non-Hispanic Black | 15 (26.32) | 49 (22.90) |  |  |  |
|  | Non-Hispanic Asian | 9 (15.79) | 23 (10.75) |  |  |  |
|  | Other Race - Including Multi-Racial | 1 (1.75) | 15 (7.01) |  |  |  |
| **Education** (%) | Less than 9th grade | 10 (17.54) | 32 (14.95) | 0.9082 |  |  |
|  | 9-11th grade (Includes 12th grade with no diploma) | 8 (14.04) | 24 (11.21) |  |  |  |
|  | High school graduate/GED or equivalent | 12 (21.05) | 51 (23.83) |  |  |  |
|  | Some college or AA degree | 18 (31.58) | 62 (28.97) |  |  |  |
|  | College graduate or above | 9 (15.79) | 44 (20.56) |  |  |  |
|  | Don't know | 0 (0.00) | 1 (0.47) |  |  |  |
| **High Blood Pressure** (%) | Yes | 40 (70.18) | 136 (63.55) | 0.3438 |  |  |
|  | No | 16 (28.07) | 77 (35.98) |  |  |  |
|  | Don't know | 1 (1.75) | 1 (0.47) |  |  |  |
| **High Cholesterol Level** (%) | Yes | 37 (64.91) | 122 (57.01) | 0.0202 |  |  |
|  | No | 17 (29.82) | 91 (42.52) |  |  |  |
|  | Refused | 1 (1.75) | 0 (0.00) |  |  |  |
|  | Don't know | 2 (3.51) | 1 (0.47) |  |  |  |
| **Age had diabetes** (mean (SD)) |  | 49.351 (14.785) | 64.238 (112.654) | 0.3211 |  |  |
| **Taking Insulin** (%) | Yes | 29 (50.88) | 46 (21.50) | <0.0001 |  |  |
|  | No | 28 (49.12) | 168 (78.50) |  |  |  |
| **Past 12 month how often have alcohol drink** (%) | Never in the last year | 32 (56.14) | 91 (42.52) | 0.7144 |  |  |
|  | Every day | 0 (0.00) | 2 (0.93) |  |  |  |
|  | Nearly every day | 1 (1.75) | 6 (2.80) |  |  |  |
|  | 3 to 4 times a week | 0 (0.00) | 7 (3.27) |  |  |  |
|  | 2 times a week | 2 (3.51) | 10 (4.67) |  |  |  |
|  | Once a week | 1 (1.75) | 12 (5.61) |  |  |  |
|  | 2 to 3 times a month | 4 (7.02) | 13 (6.07) |  |  |  |
|  | Once a month | 4 (7.02) | 19 (8.88) |  |  |  |
|  | 7 to 11 times in the last year | 3 (5.26) | 7 (3.27) |  |  |  |
|  | 3 to 6 times in the last year | 4 (7.02) | 18 (8.41) |  |  |  |
|  | 1 to 2 times in the last year | 6 (10.53) | 29 (13.55) |  |  |  |
| **URXUCR** (mean (SD)) |  | 98.895 (65.499) | 124.603 (83.474) | 0.0321 |  |  |
| **URXCNP** (mean (SD)) |  | 1.645 (3.047) | 1.967 (3.975) | 0.5707 |  |  |
| **URXCOP** (mean (SD)) |  | 10.554 (20.664) | 12.596 (32.645) | 0.6541 |  |  |
| **URXECP** (mean (SD)) |  | 15.540 (18.254) | 18.749 (37.665) | 0.5336 |  |  |
| **URXMBP** (mean (SD)) |  | 9.760 (7.953) | 14.569 (18.953) | 0.0625 |  |  |
| **URXMC1** (mean (SD)) |  | 3.049 (10.941) | 12.482 (148.306) | 0.6322 |  |  |
| **URXMEP** (mean (SD)) |  | 588.910 (1859.299) | 454.036 (1898.260) | 0.6325 |  |  |
| **URXMHH** (mean (SD)) |  | 12.284 (19.502) | 10.926 (16.740) | 0.5999 |  |  |
| **URXMIB** (mean (SD)) |  | 7.711 (6.632) | 10.192 (11.019) | 0.1058 |  |  |
| **URXMOH** (mean (SD)) |  | 5.004 (6.169) | 5.525 (9.414) | 0.6926 |  |  |
| **URXMZP** (mean (SD)) |  | 6.290 (7.742) | 7.086 (8.568) | 0.5257 |  |  |
