# Supplementary material for: From risk factors to molecular targets: clinical associations and molecular docking insights into phthalate-associated diabetic retinopathy
Source: Front Med (Lausanne). 2026 May 13;13:1792532. doi: 10.3389/fmed.2026.1792532 (PMC13212054; doi:10.3389/fmed.2026.1792532)
Supplement: Supplementary file 9 [file Table_5.docx]

Supplementary Table 5. Distribution of Phthalate metabolites.

| **Abbreviation** | **Mean** | **SD** | **Min, Max** | **5th** | **25th** | **50th** | **75th** | **95th** |
| --- | --- | --- | --- | --- | --- | --- | --- | --- |
| MCNP | 2.528 | 5.036 | 0.14,45.8 | 0.3 | 0.7 | 1.1 | 2.4 | 7.9 |
| MCOP | 15.731 | 42.400 | 0.21,351.7 | 1.1 | 2.6 | 5.4 | 10.6 | 59.6 |
| MECPP | 19.336 | 39.646 | 0.8,303.8 | 1.85 | 4.85 | 8.8 | 17.85 | 58.45 |
| MnBP | 13.804 | 17.294 | 0.28,137.4 | 0.85 | 4.8 | 9.3 | 14.9 | 39 |
| MCPP | 26.449 | 227.33 | 0.28,2170 | 0.28 | 0.55 | 1.1 | 2 | 6.8 |
| MEP | 241.13 | 1076.3 | 0.85,12885 | 3.45 | 11.95 | 28.4 | 70.5 | 1026.45 |
| MEHHP | 8.8630 | 13.031 | 0.28,113.1 | 1.05 | 2.6 | 5 | 8.95 | 32.6 |
| MiBP | 11.606 | 13.042 | 0.57,83.9 | 1.35 | 3.9 | 7.3 | 13.75 | 37.05 |
| MEOHP | 5.971 | 9.715 | 0.3,86.9 | 0.4 | 1.5 | 3.3 | 6.05 | 21 |
| MBzP | 5.981 | 7.486 | 0.21,45 | 0.4 | 1.4 | 2.8 | 7.9 | 21.85 |
