# Supplementary material for: From risk factors to molecular targets: clinical associations and molecular docking insights into phthalate-associated diabetic retinopathy
Source: Front Med (Lausanne). 2026 May 13;13:1792532. doi: 10.3389/fmed.2026.1792532 (PMC13212054; doi:10.3389/fmed.2026.1792532)
Supplement: Supplementary file 10 [file Table_6.docx]

**Supplementary Figure 1**: Feature selection and risk factor analysis for diabetes in the self-collected clinical cohort.

1. Variable importance from the random forest model.
2. Multivariate logistic regression results for diabetes risk.
3. LASSO regression coefficient profiles and cross-validation.
4. Risk factors for diabetes identified by LASSO regression.

**Supplementary Figure 2**: Distribution of urinary phthalate metabolite concentrations. (A) Mono (5-carboxy-2-ethylpentyl) phthalate (MECPP); (B) Monobutyl phthalate (MNBP); (C) Mono-(3-carboxypropyl) phthalate (MCPP); (D) Mono-ethyl phthalate (MEP).

**Supplementary Figure 3**: Distribution of urinary phthalate metabolite concentrations. (A) Mono-(2-ethyl-5-hydroxyhexyl) phthalate (MEHP); (B) Mono-(2-ethyl-5-oxohexyl) phthalate (MEOH); (C) Mono-benzyl phthalate (MBZP); (D) Mono(carboxynonyl) phthalate (MNOP); (E) Mono(carboxyoctyl) phthalate (MCOP).

**Supplementary Figure 4**: **Logistic regression was used to examine the association between quartiles of individual urinary phthalate metabolites and diabetic retinopathy across three adjustment models.** For each metabolite, results are presented for Model 1 (unadjusted; left panel), Model 2 (adjusted for gender, race, and age; middle panel), and Model 3 (further adjusted for education level and alcohol use; right panel):

(A) log10-transformed MBzP

(B) log10-transformed MCNP

(C) log10-transformed MCOP

(D) log10-transformed MECPP

(E) log10-transformed MNBP

(F) log10-transformed MCPP

(G) log10-transformed MEP

(H) log10-transformed MIBP

(I) log10-transformed MEHP

(J) log10-transformed MEOH
